# Supplementary material for: Clinical phenotypes and outcomes associated with SARS-CoV-2 variant Omicron in critically ill French patients with COVID-19
Source: Nat Commun. 2022 Oct 12;13:6025. doi: 10.1038/s41467-022-33801-z (PMC9555693; doi:10.1038/s41467-022-33801-z)
Supplement: Supplementary file 1 — Supplementary information [file 41467_2022_33801_MOESM1_ESM.pdf]

Clinical phenotypes and outcomes associated with SARS-CoV-2 variant Omicron, sublineages BA.1, BA.1.1, BA.2 in critically ill French patients with COVID-19

Author list

Nicolas de Prost, Etienne Audureau, Nicholas Heming, Elyanne Gault, Tài Pham, Amal Chaghouri, Nina de Montmollin, Guillaume Voiriot, Laurence Morand-Joubert, Adrien Joseph, Marie-Laure Chaix, Sébastien Préau, Raphaël Favory, Aurélie Guigon, Charles-Edouard Luyt, Sonia Burrel, Julien Mayaux, Stéphane Marot, Damien Roux, Diane Descamps, Sylvie Meireles, Frédéric Pène, Flore Rozenberg, Damien Contou, Amandine Henry, Stéphane Gaudry, Ségolène Brichler, Jean-François Timsit, Antoine Kimmoun, Cédric Hartard, Louise-Marie Jandeau, Samira Fafi-Kremer, Paul Gabarre, Malo Emery, Claudio Garcia-Sanchez, Sébastien Jochmans, Aurélia Pitsch, Djillali Annane, Elie Azoulay, Armand Mekontso Dessap, Christophe Rodriguez , Jean-Michel Pawlotsky, Slim Fourati

Supplementary Information

Content

Supplementary Table 1……………………………………………………………….Page 2

Supplementary Table 2……………………………………………………………….Page 4

Supplementary Table 3……………………………………………………………….Page 6

Supplementary Table 4……………………………………………………………….Page 7

Supplementary Table 1. Association of variables available within 24 hours of ICU admission and day-28 mortality by univariable logistic regression analysis in patients with Delta and Omicron infection

		Day-28 survivors N=175	Day-28 non-survivors N=84	OR (CI95%)	p-value
Demographics and comorbidities					
Sex, females		58 (33.14%)	24 (28.57%)	0.81 (0.46;1.42)	0,459
Age, years		58.53 (±12.73)	66.70 (±11.30)	1.06 (1.04;1.09)	**<0,0001**
Diabetes		51 (29.14%)	35 (42.68%)	1.81 (1.05;3.12)	**0,033**
Obesity		71 (41.04%)	28 (33.33%)	0.72 (0.42;1.24)	0,235
Chronic heart failure		8 (4.57%)	13 (15.66%)	3.88 (1.54;9.77)	**0,004**
Hypertension		71 (40.57%)	47 (56.63%)	1.91 (1.13;3.24)	**0,016**
Chronic respiratory failure		14 (8.00%)	9 (10.84%)	1.40 (0.58;3.38)	0,456
Chronic renal failure		23 (13.22%)	23 (28.05%)	2.56 (1.33;4.91)	**0,005**
Cirrhosis		3 (1.71%)	1 (1.20%)	0.70 (0.07;6.82)	0,758
Immunosuppression		43 (24.57%)	36 (43.37%)	2.35 (1.35;4.09)	**0,002**
Immunosuppression (3 cat.)	None	132 (75.43%)	47 (56.63%)	1(ref)	**0,027**
	Solid organ transplant	22 (12.57%)	18 (21.69%)	2.30 (1.13;4.66)	**0,021**
	Onco-hematological malignar	11 (6.29%)	10 (12.05%)	2.55 (1.02;6.40)	**0,046**
	Others[a]	10 (5.71%)	8 (9.64%)	2.25 (0.84;6.03)	0,108
Number of comorbidities		1 (1;2)	2 (1;3)	1.41 (1.16;1.70)	**0,0004**
Clinical frailty scale		3 (2;3)	3 (3;4)	1.30 (1.09;1.55)	**0,003**
SARS-CoV-2 infection and Vaccination					
Clade	Delta	79 (45.14%)	32 (38.10%)	1(ref)	0,284
	Omicron	96 (54.86%)	52 (61.90%)	1.34 (0.79;2.28)	0,284
Previous SARS-CoV-2 infection		13 (7.47%)	3 (3.61%)	0.46 (0.13;1.68)	0,242
SARS-CoV-2 vaccination (binaire)		70 (40.23%)	51 (60.71%)	2.30 (1.35;3.91)	**0,002**
Number of doses among vaccinated		3 (2;3)	3 (2;3)	0.81 (0.45;1.48)	0,494
1st dose - ICU admission[b], days		24 (17;62)	159 (11;307)	1.01 (0.99;1.04)	0,360
2nd dose - ICU admission[b], days		157 (57;211)	174 (120;241)	1.00 (0.99;1.01)	0,388
3rd dose - ICU admission[b], days		140 (34;217)	100 (46;179)	1.00 (0.99;1.01)	0,570
Last dose - ICU admission[b], days		131.50 (34;214)	135 (48;202)	1.00 (1.00;1.00)	0,910
SARS-CoV-2 serology at ICU a	Unavailable	65 (37.14%)	31 (36.90%)	1(ref)	0,334
	Negative	57 (32.57%)	34 (40.48%)	1.25 (0.68;2.29)	0,467
	Positive	53 (30.29%)	19 (22.62%)	0.75 (0.38;1.48)	0,408
First symptoms - ICU admission, days		9 (6;11)	7.50 (5;10)	0.98 (0.93;1.03)	0,478
SARS-CoV-2 RNA detection in nasopharyngeal swabs, Ct		24 (20.50;27.50)	20 (18;23)	0.82 (0.75;0.90)	**<0,0001**
Patients severity upon ICU admission and biological features					
WHO 10-point scale		6 (6;6)	6 (6;8)	1.17 (0.91;1.50)	0,210
SAPS II score		31 (24;39)	40 (31;51)	1.05 (1.03;1.08)	**<0,0001**
SOFA score		4 (2;5)	5 (3.50;7)	1.21 (1.09;1.34)	**0,0003**
PaO2/FiO2 ratio, mmHg		124 (80;178)	85 (64;133)	1.00 (0.99;1.00)	0,066
Arterial lactate level, mM		1.50 (1.10;2.10)	1.90 (1.15;2.40)	1.05 (0.92;1.19)	0,467
Blood leukocytes, G/L		8.35 (5.60;12.50)	8.00 (4.65;11.65)	1.01 (0.97;1.04)	0,728
Blood lymphocytes, G/L		0.60 (0.40;0.90)	0.40 (0.20;0.70)	1.02 (0.96;1.08)	0,527
Blood platelets, G/L		239.50 (180;316)	179 (128;254)	0.99 (0.99;1.00)	**<0,0001**
Serum urea level, mM		7 (5;12)	12 (7.50;19.5)	1.06 (1.03;1.09)	**0,0001**
Serum creatinine level, µM		75 (59;110)	107 (70;166.5)	1.00 (1.00;1.01)	**0,006**
Bacterial coinfection		23 (13.14%)	13 (15.48%)	1.21 (0.58;2.53)	0,612
Pulmonary embolism		11 (6.51%)	5 (5.95%)	0.91 (0.31;2.71)	0,864
Lung parenchyma involvement, %		50 (50;75)	50 (40;75)	1.01 (0.99;1.02)	0,470
Oxygen/ventilatory support	Oxygen	34 (19.43%)	10 (11.90%)	1(ref)	0,404
	High flow oxygen	90 (51.43%)	43 (51.19%)	1.62 (0.73;3.59)	0,231
	NIV/C-PAP	13 (7.43%)	8 (9.52%)	2.09 (0.68;6.47)	0,200
	Invasive MV	38 (21.71%)	23 (27.38%)	2.06 (0.86;4.94)	0,106
ECMO		9 (5.20%)	2 (2.38%)	0.44 (0.09;2.10)	0,307
Vasopressor support		18 (10.71%)	15 (18.07%)	1.84 (0.87;3.86)	0,108

Results are N (%), means (±standard deviation) or medians (interquartile range, i.e., quartile 1;quartile 3). [a]includes HIV infection, long-term corticosteroid treatment, and other immunosuppressive treatments; [b]time lag between the last vaccination dose and ICU admission; [c]defined as < 30 Binding Antibody Units (BAU)/mL; ICU: intensive care unit; Ct: cycle threshold; WHO: World Health Organization; SOFA: Sequential Organ Failure Assessment; SAPS II: Simplified Acute Physiology Score II; NIV: non-invasive ventilation; C-PAP; Continuous-positive airway pressure; MV: mechanical ventilation; ECMO:

extracorporeal mechanical ventilation; Two-tailed p-values come from unadjusted comparisons using Chi square or Fisher's exact tests for categorical variables, and t-tests or Mann-Whitney tests for continuous variables, as appropriate. No adjustment for multiple comparisons was performed; **Bolded** p-values are significant at the $p<0.05$ level.

Supplementary Table 2. Association of variables available within 24 hours of ICU admission and day-28 mortality by univariable logistic regression analysis in patients with Omicron infection

		Day-28 survivors N=96	Day-28 non-survivors N=52	OR (CI95%)	p-value
Demographics and comorbidities					
Sex, females		30 (31.25%)	12 (23.08%)	0.66 (0.30;1.43)	0,294
Age, years		62.02 (±11.33)	67.28 (±8.73)	1.05 (1.02;1.09)	**0,006**
Diabetes		27 (28.13%)	24 (47.06%)	2.27 (1.12;4.61)	**0,023**
Obesity		35 (37.23%)	16 (30.77%)	0.75 (0.36;1.54)	0,433
Chronic heart failure		6 (6.25%)	9 (17.31%)	3.14 (1.05;9.38)	**0,041**
Hypertension		48 (50.00%)	28 (53.85%)	1.17 (0.59;2.29)	0,655
Chronic respiratory failure		12 (12.50%)	7 (13.46%)	1.09 (0.40;2.96)	0,867
Chronic renal failure		17 (17.89%)	17 (33.33%)	2.29 (1.05;5.02)	**0,038**
Cirrhosis		2 (2.08%)	1 (1.92%)	0.92 (0.08;10.41)	0,947
Immunosuppression		34 (35.42%)	30 (57.69%)	2.49 (1.25;4.96)	**0,010**
Immunosuppression (3 cat.)	None	62 (64.58%)	22 (42.31%)	1(ref)	0,063
	Solid organ transplant	17 (17.71%)	15 (28.85%)	2.49 (1.07;5.80)	**0,035**
	Onco-hematological malignan(	9 (9.38%)	10 (19.23%)	3.13 (1.13;8.71)	**0,029**
	Others[a]	8 (8.33%)	5 (9.62%)	1.76 (0.52;5.96)	0,363
Number of comorbidities		2 (1;3)	2.50 (1;3.5)	1.46 (1.12;1.91)	**0,005**
Clinical frailty scale		3 (2;4)	3 (3;4)	1.40 (1.10;1.80)	**0,007**
SARS-CoV-2 infection and Vaccination					
Omicron - variant	Omicron - BA.1.1, BA.1 ou B.1	68 (80.95%)	41 (89.13%)	1(ref)	0,231
	Omicron - BA.2	16 (19.05%)	5 (10.87%)	0.52 (0.18;1.52)	0,231
Previous SARS-CoV-2 infection		3 (3.16%)	3 (5.77%)	1.88 (0.37;9.65)	0,451
SARS-CoV-2 vaccination (binaire)		52 (54.17%)	37 (71.15%)	2.09 (1.01;4.30)	**0,046**
Number of doses among vaccinated		3 (2;3)	3 (2;3)	0.84 (0.39;1.82)	0,660
1st dose - ICU admission[b], days		17 (17;17)	307 (307;307)		
2nd dose - ICU admission[b], days		157 (57;211)	169 (125;241)	1.00 (0.99;1.01)	0,761
3rd dose - ICU admission[b], days		154.5 (81.5;231.5)	100 (48;179)	1.00 (0.99;1.00)	0,291
Last dose - ICU admission[b], days		157 (70;217)	146 (49;202)	1.00 (0.99;1.00)	0,563
SARS-CoV-2 serology at ICU ad	Unavailable	41 (42.71%)	20 (38.46%)	1(ref)	0,844
	Negative	31 (32.29%)	17 (32.69%)	1.12 (0.51;2.50)	0,774
	Positive	24 (25.00%)	15 (28.85%)	1.28 (0.55;2.96)	0,562
First symptoms - ICU admission, days		7 (5;10)	7 (4;11)	1.01 (0.95;1.07)	0,838
SARS-CoV-2 RNA detection in nasopharyngeal swabs, Ct		25 (20;27)	20 (18;24)	0.83 (0.74;0.93)	**0,0008**
Patients severity upon ICU admission and biological features					
WHO 10-point scale		6 (6;6)	6 (6;7.5)	1.13 (0.81;1.58)	0,466
SAPS II score		33 (27;41)	42 (32;52)	1.06 (1.03;1.09)	**0,0003**
SOFA score		4 (3;6)	6 (4;7)	1.25 (1.09;1.44)	**0,002**
PaO2/FiO2 ratio, mmHg		124 (86;178)	89 (70;120)	0.99 (0.98;1.00)	**0,005**
Arterial lactate level, mM		1.50 (1.10;2)	2 (1;2.50)	1.13 (0.91;1.40)	0,268
Blood leukocytes, G/L		7.70 (5.05;11.45)	8.20 (4.35;13.05)	1.04 (0.99;1.09)	0,162
Blood lymphocytes, G/L		0.50 (0.40;0.80)	0.40 (0.20;0.70)	1.04 (0.96;1.13)	0,353
Blood platelets, G/L		203 (157.50;291)	154.50 (114;244)	0.99 (0.99;1.00)	**0,009**
Serum urea level, mM		8 (5;13.5)	12.50 (8;21.5)	1.04 (1.01;1.08)	**0,014**
Serum creatinine level, µM		89 (63;132)	110.50 (80.50;191.5)	1.00 (1.00;1.01)	0,100
Bacterial coinfection		10 (10.42%)	11 (21.15%)	2.31 (0.91;5.87)	0,079
Pulmonary embolism		4 (4.26%)	5 (9.62%)	2.39 (0.61;9.34)	0,209
Lung parenchyma involvement, %		50 (45;75)	50 (40;75)	1.00 (0.98;1.02)	0,873
Oxygen/ventilatory support	Oxygen	18 (18.75%)	9 (17.31%)	1(ref)	0,612
	High flow oxygen	51 (53.13%)	25 (48.08%)	0.98 (0.39;2.49)	0,967
	NIV/C-PAP	10 (10.42%)	4 (7.69%)	0.80 (0.20;3.27)	0,756
	Invasive MV	17 (17.71%)	14 (26.92%)	1.65 (0.57;4.79)	0,360
ECMO		1 (1.05%)	1 (1.92%)	1.84 (0.11;30.09)	0,668
Vasopressor support		8 (8.79%)	11 (21.57%)	2.85 (1.06;7.65)	**0,037**

Results are N(%), means (±standard deviation) or medians (interquartile range, i.e., quartile 1;quartile 3). [a]includes HIV infection, long-term corticosteroid treatment, and other immunosuppressive treatments; [b]time lag between the last vaccination dose and ICU admission; [c]defined as <30 Binding Antibody Units (BAU)/mL; ICU: intensive care unit; Ct: cycle threshold; WHO: World Health Organization; SOFA: Sequential Organ Failure Assessment; SAPS II: Simplified Acute Physiology Score II; NIV: non-invasive ventilation; C-PAP; Continuous-positive airway pressure; MV: mechanical ventilation; ECMO: extracorporeal mechanical ventilation; Two-tailed p-values come from unadjusted comparisons using Chi square or Fisher's

exact tests for categorical variables, and t-tests or Mann-Whitney tests for continuous variables, as appropriate. No adjustment for multiple comparisons was performed; **Bolded** p-values are significant at the p<0.05 level.

Supplementary Table 3. Independent predictors of day-28 mortality available within 24 hours after ICU admission by multivariable logistic regression analysis in patients infected with variant Omicron (n=130).

		**aOR (CI 95%)**	**p-value**
Age, years		1.06 (1.01;1.11)	**0.030**
SOFA score		1.29 (1.09;1.53)	**0.004**
Omicron - variant	Omicron - BA.1, BA.1.1	1(ref)	
	Omicron - BA.2	0.35 (0.10;1.18)	0.091
Chronic heart failure		9.40 (1.92;46.10)	**0.006**
Diabetes		2.44 (1.00;5.93)	**0.049**
Sex, females		0.51 (0.18;1.44)	0.202
Immunosuppression		1.64 (0.59;4.56)	0.343
SARS-CoV-2 vaccination		1.65 (0.56;4.86)	0.361

aOR (CI 95%): adjusted Odds Ratio (95% confidence interval) ; SOFA: Sequential Organ Failure Assessment; p-values come from multivariable logistic regression models; **Bolded** p-values are significant at the $p<0.05$ level.

Supplementary Table 4. Comparison of patients with severe SARS-CoV-2 infection at the time of ICU admission according to SARS-CoV-2 Omicron identified variants (N=130)

		**Omicron - BA.1, BA.1.1**<br>N=109	**Omicron - BA.2**<br>N=21	**p-value**
Demographics and comorbidities				
Sex, females		32 (29.36%)	5 (23.81%)	0,606
Age, years		64.3 (±10.6)	64.4 (±9.1)	0,941
Diabetes		42 (38.89%)	7 (33.33%)	0,631
Obesity		36 (33.33%)	6 (30.00%)	0,771
Chronic heart failure		12 (11.01%)	2 (9.52%)	0,841
Hypertension		58 (53.21%)	12 (57.14%)	0,741
Chronic respiratory failure		6 (5.50%)	8 (38.10%)	**0,000**
Chronic renal failure		28 (26.17%)	5 (23.81%)	0,821
Cirrhosis		2 (1.83%)	0 (0.00%)	0,532
Immunosuppression		46 (42.20%)	12 (57.14%)	0,207
Immunosuppression (3 cat.)	None	63 (57.80%)	9 (42.86%)	0,257
	Solid organ transplant	25 (22.94%)	6 (28.57%)	
	Onco-hematological malignancies	11 (10.09%)	5 (23.81%)	
	Others[a]	10 (9.17%)	1 (4.76%)	
Number of comorbidities		2 (1;3)	2 (2;3)	0,193
Clinical frailty scale		3 (3;4)	3 (3;4)	0,489
SARS-CoV-2 infection and Vaccination				
Previous SARS-CoV-2 infection		4 (3.70%)	1 (4.76%)	0,818
SARS-CoV-2 vaccination		64 (58.72%)	18 (85.71%)	**0,019**
Number of doses among vaccinated		3 (2;3)	3 (3;3)	0,059
1st dose - ICU admission[b], days		162 (17;307)	.	
2nd dose - ICU admission[b], days		174 (97;226)	.	
3rd dose - ICU admission[b], days		134 (48;217)	153 (144;163)	0,929
Last dose - ICU admission[b], days		157 (57;241)	153 (144;163)	0,908
SARS-CoV-2 serology at ICU admission	Unavailable	46 (42.20%)	7 (33.33%)	**0,016**
	Negative	38 (34.86%)	3 (14.29%)	
	Positive	25 (22.94%)	11 (52.38%)	
First symptoms - ICU admission, days		7 (5;10)	7 (3;11)	0,505
SARS-CoV-2 RNA detection in nasopharyngeal swabs, Ct		22 (19;26)	19 (18;22)	0,085
Patients severity upon ICU admission and biological features				
WHO 10-point scale		6 (6;6)	6 (6;8)	0,205
SAPS II score		34 (27;45)	39 (33;42)	0,192
SOFA score		4 (3;6)	5 (4;7)	0,259
PaO_2/FiO_2 ratio, mmHg		103 (76;142)	145 (111;170)	**0,017**
Arterial lactate level, mM		1.6 (1.1;2.1)	1.5 (1.0;2.4)	0,903
Blood leukocytes, G/L		7.7 (4.8;11.3)	10.0 (4.1;17.5)	0,239
Blood lymphocytes, G/L		0.5 (0.3;0.8)	0.4 (0.2;0.6)	0,316
Blood platelets, G/L		198 (141;284)	146 (111;220)	**0,028**
Serum urea level, mM		9 (6;16)	12 (7;17)	0,226
Serum creatinine level, µM		94 (67;158)	110 (88;177)	0,237
Bacterial coinfection		13 (11.93%)	6 (28.57%)	**0,048**

Pulmonary embolism		7 (6.54%)	1 (4.76%)	0,758
Lung parenchyma involvement, %		50 (40;75)	62 (43;75)	0,722
Oxygen/ventilatory support	Oxygen	22 (20.18%)	4 (19.05%)	0,512
	High flow oxygen	57 (52.29%)	8 (38.10%)	
	NIV/C-PAP	8 (7.34%)	3 (14.29%)	
	Invasive MV	22 (20.18%)	6 (28.57%)	
ECMO		1 (0.92%)	1 (4.76%)	0,190
Vasopressor support		14 (13.46%)	4 (19.05%)	0,000
Management and outcomes during ICU stay				
Invasive MV		53 (48.62%)	9 (42.86%)	0.628
Prone positioning		40 (40.40%)	6 (30.00%)	0.457
MV duration, days		16 (7;23)	9 (4;14)	0.073
ECMO support		8 (7.34%)	1 (4.76%)	1.000
Duration of ECMO, days		8 (8;25)	-	-
Vasopressor support		50 (43.30%)	5 (23.81%)	0.057
Duration of vasopressors, days		5 (1;13)	4 (2;10)	0.563
Renal Replacement Therapy		21 (19.27%)	2 (9.52%)	0.365
Ventilator-acquired pneumonia		35 (32.71%)	3 (14.29%)	0.119
Ventilator-acquired pneumonia (among IMV)		35 (68.63%)	3 (33.33%)	0.063
CAPA		4 (3.74%)	2 (9.52%)	0.255
Dexamethasone		91 (89.22%)	15 (71.43%)	**0.031**
Tocilizumab		41 (40.20%)	3 (14.29%)	**0.026**
Monoclonal antibodies		20 (20.00%)	3 (14.29%)	0.762
Casirivimab - Imdevimab		20 (100.00%)	3 (100.00%)	-
Tixagevimab - Cilgavimab		16 (80.00%)	3 (100.00%)	1.000
Sotrovimab		4 (20.00%)	0 (0.00%)	1.000
Day-28 mortality		41 (37.61%)	5 (23.81%)	0.230

Results are N (%), means (±standard deviation) or medians (interquartile range, *i.e., quartile 1;quartile 3*). [a]includes HIV infection, long-term corticosteroid treatment, and other immunosuppressive treatments; [b]time lag between the last vaccination dose and ICU admission; [c]defined as <30 Binding Antibody Units (BAU)/mL; ICU: intensive care unit; Ct: cycle threshold; WHO: World Health Organization; SOFA: Sequential Organ Failure Assessment; SAPS II: Simplified Acute Physiology Score II; NIV: non-invasive ventilation; C-PAP; Continuous-positive airway pressure; MV: mechanical ventilation; ECMO: extracorporeal mechanical ventilation; Two-tailed p-values come from unadjusted comparisons using Chi square or Fisher's exact tests for categorical variables, and t-tests or Mann-Whitney tests for continuous variables, as appropriate. No adjustment for multiple comparisons was performed; **Bolded** p-values are significant at the p<0.05 level.
