# Supplementary material for: Clinical phenotypes and outcomes associated with SARS-CoV-2 variant Omicron in critically ill French patients with COVID-19
Source: Nat Commun. 2022 Oct 12;13:6025. doi: 10.1038/s41467-022-33801-z (PMC9555693; doi:10.1038/s41467-022-33801-z)
Supplement: Supplementary file 2 — Reporting Summary [file 41467_2022_33801_MOESM2_ESM.pdf]

nature portfolio

Corresponding author(s): FOURATI Slim

Last updated by author(s): Sep 2, 2022

Reporting Summary

Nature Portfolio wishes to improve the reproducibility of the work that we publish. This form provides structure for consistency and transparency in reporting. For further information on Nature Portfolio policies, see our Editorial Policies and the Editorial Policy Checklist.

Statistics

For all statistical analyses, confirm that the following items are present in the figure legend, table legend, main text, or Methods section.

n/a	Confirmed	
☐	☒	The exact sample size (n) for each experimental group/condition, given as a discrete number and unit of measurement
☐	☒	A statement on whether measurements were taken from distinct samples or whether the same sample was measured repeatedly
☐	☒	The statistical test(s) used AND whether they are one- or two-sided<br>*Only common tests should be described solely by name; describe more complex techniques in the Methods section.*
☐	☒	A description of all covariates tested
☐	☒	A description of any assumptions or corrections, such as tests of normality and adjustment for multiple comparisons
☐	☒	A full description of the statistical parameters including central tendency (e.g. means) or other basic estimates (e.g. regression coefficient) AND variation (e.g. standard deviation) or associated estimates of uncertainty (e.g. confidence intervals)
☐	☒	For null hypothesis testing, the test statistic (e.g. F, t, r) with confidence intervals, effect sizes, degrees of freedom and P value noted<br>*Give P values as exact values whenever suitable.*
☒	☐	For Bayesian analysis, information on the choice of priors and Markov chain Monte Carlo settings
☒	☐	For hierarchical and complex designs, identification of the appropriate level for tests and full reporting of outcomes
☒	☐	Estimates of effect sizes (e.g. Cohen's d, Pearson's r), indicating how they were calculated

Our web collection on statistics for biologists contains articles on many of the points above.

Software and code

Policy information about availability of computer code

Data collection	Data were collected using the CleanWeb e-platform for clinical trials (Telemedicine Technologies)
Data analysis	Analyses were performed using Stata V16.1 statistical software (StataCorp, College Station, TX, USA), and R 4.2.0 (R Foundation for Statistical Computing, Vienna, Austria)

For manuscripts utilizing custom algorithms or software that are central to the research but not yet described in published literature, software must be made available to editors and reviewers. We strongly encourage code deposition in a community repository (e.g. GitHub). See the Nature Portfolio guidelines for submitting code & software for further information.

Data

Policy information about availability of data

All manuscripts must include a data availability statement. This statement should provide the following information, where applicable:

- Accession codes, unique identifiers, or web links for publicly available datasets
- A description of any restrictions on data availability
- For clinical datasets or third party data, please ensure that the statement adheres to our policy

All SARS CoV-2 GENBANK accession code sequences are available.

Human research participants

Policy information about studies involving human research participants and Sex and Gender in Research.

Reporting on sex and gender	Sex was determined based on self reporting
Population characteristics	Patients had the following inclusion criteria: age ≥18 years, SARS-CoV-2 infection confirmed by a positive reverse transcriptase-polymerase chain reaction (RT-PCR) in nasopharyngeal swab samples, admission in the ICU for acute respiratory failure (i.e., peripheral oxygen saturation (SpO2) ≤90% and need for supplemental oxygen or any kind of ventilator support).
Recruitment	This is a prospective multicenter observational cohort study. Consecutive patients admitted between December 7th , 2021 and May 1st, 2022 in one of the 20 participating ICUs were eligible for inclusion in the SEVARVIR cohort study (NCT05162508) if they presented inclusion criteria. The study only included patients admitted in the intensive care unit and thus did not include less severe patients (not hospitalized or hospitalized in conventional units). The study population thus selected a subgroup of severe, critically ill, COVID-19 patients.
Ethics oversight	The study was approved by the Comité Ethique de Protection des Personnes Sud-Méditerranée I (N° EudraCT/ID-RCB: 2021-A02914-37). Informed consent was obtained from all patients or their relatives.

Note that full information on the approval of the study protocol must also be provided in the manuscript.

Field-specific reporting

Please select the one below that is the best fit for your research. If you are not sure, read the appropriate sections before making your selection.

☒ Life sciences ☐ Behavioural & social sciences ☐ Ecological, evolutionary & environmental sciences

For a reference copy of the document with all sections, see nature.com/documents/nr-reporting-summary-flat.pdf

Life sciences study design

All studies must disclose on these points even when the disclosure is negative.

Sample size	No a priori sample size calculation was made for this study. We included all patients with SARS COV-2 infection AND acute respiratory failure who were hospitalised in participating Intensive Care Units (n=20) during the fifth and sixth COVID-19 epidemic waves in France (December-2021-May 2022).
Data exclusions	Patients with SARS COV-2 infection hospitalised in Intensive Care Units WITHOUT acute respiratory failure were excluded. No data were excluded from the analysis.
Replication	Source data are provided with this article. Pseudonymized patient data may be available upon written reasonable request to the corresponding author (S.F.).
Randomization	N/A
Blinding	N/A

Reporting for specific materials, systems and methods

We require information from authors about some types of materials, experimental systems and methods used in many studies. Here, indicate whether each material, system or method listed is relevant to your study. If you are not sure if a list item applies to your research, read the appropriate section before selecting a response.

Materials & experimental systems

n/a	Involved in the study	
☒	☐	Antibodies
☒	☐	Eukaryotic cell lines
☒	☐	Palaeontology and archaeology
☒	☐	Animals and other organisms
☐	☒	Clinical data
☒	☐	Dual use research of concern

Methods

n/a	Involved in the study	
☒	☐	ChIP-seq
☒	☐	Flow cytometry
☒	☐	MRI-based neuroimaging

Clinical data

Policy information about clinical studies
All manuscripts should comply with the ICMJEguidelines for publication of clinical research and a completedCONSORT checklist must be included with all submissions.

Clinical trial registration	NCT05162508
Study protocol	SEVARVIR cohort study
Data collection	Patients admitted between December 7th , 2021 (week 49/21) and May 1st, 2022 (week 17/22) in one of the 20 participating ICUs (17 from the Greater Paris area and 3 from the North-East of France) were included
Outcomes	The primary outcome measure of the study was day-28 mortality.<br>Secondary outcome measures included organ failures (need for invasive mechanical ventilation, extracorporeal mechanical ventilation, vasopressor support or renal replacement therapy) during intensive care unit (ICU) stay, development of ventilator-acquired pneumonia and COVID-19-associated pulmonary aspergillosis, management of COVID-19 and duration of ICU stay.
